# Capsule Endoscopy to Detect Normally Positioned Duodenal Papilla: Performance Comparison of SB and SB2

**DOI:** 10.1155/2012/202935

**Published:** 2012-04-04

**Authors:** Sanghoon Park, Hoon Jai Chun, Bora Keum, Yeon Seok Seo, Yong Sik Kim, Yoon Tae Jeen, Hong Sik Lee, Soon Ho Um, Chang Duck Kim, Ho Sang Ryu

**Affiliations:** Division of Gastroenterology and Hepatology, Department of Internal Medicine, Institute of Digestive Disease and Nutrition, Korea University College of Medicine, Korea University Medical Center, Seoul 136-705, Republic of Korea

## Abstract

*Purpose*. PillCam SB2 capsule endoscopy, an upgraded version of widely used SB capsule endoscopy, was examined for its performance by comparing with SB. *Methods*. Examinees with various indications were enrolled for SB2 capsule endoscopy; subjects were also enlisted for the old SB capsule endoscopy. Number of photo images containing papilla of Vater was counted. Shape of the papilla seen in each image was evaluated by scoring 3 (fully observable papilla), 2 (more than half outline), or 1 (less than half outline) points. Images obtained from SB and SB2 were also subjectively compared; resolution and brightness were scored by six experienced endoscopists. *Results*. Baseline characteristics of two study groups (*n* = 30 each) were not significantly different. Number of images of the papilla revealed to show similar results between SB (3.1 ± 1.1, range 1~5) and SB2 (3.1 ± 1.5, range 1~8) (*P* = 0.62). The maximum points of outline of papilla evaluated from each subject were also similar between two groups. New SB2 revealed to be superior to SB in terms of resolution but not significantly different in brightness. *Conclusion*. Our study showed that superiority of SB2 over SB is rather marginal on examining duodenal papilla.

## 1. Introduction

Wireless capsule endoscopy (WCE) is widely used for diagnosing various small bowel diseases. However, this convenient diagnostic tool has several inherent drawbacks; inability of biopsy, lengthy time for interpretation, lack of active control of the device that just floats about the gut in accordance with peristalsis, and so forth. Relatively narrow visual field and fixed image contrast also leads to insufficient visualization of the intestinal mucosa, hindering precise diagnosis of various endoscopic findings. In our previous report that studied the limited ability of WCE to detect duodenal papilla, we proved that a technologic improvement of WCE is essential [1]. This study additionally delivered another message, that is, true diagnostic yield of WCE is unexpectedly not that high, revealing a detection rate of only 43.6%.

PillCam SB2 video capsule is a recently released version of PillCam SB video capsule. It is characterized with a wider visual field, automatic light control, and new capsule lenses, therefore enabling improved visualization of small bowel mucosa. After release of the PillCam SB2 to the local market of Korea, authors performed an investigation of comparing images and diagnostic yield of SB2 capsule with the old model SB, using normally positioned duodenal papilla as a landmark.

## 2. Methods

PillCam SB and SB2 capsule endoscopy was each performed to examinees with various indications. Patients with following conditions were excluded: previous gastrointestinal (GI) tract surgery including resection, suspicious GI obstruction, comorbid serious cardiopulmonary or renal diseases (i.e., heart failure, acute myocardial infarction, dysrhythmia, renal insufficiency, etc.), and refusal of participation. Subjects were all verified that no current medication was being administered such as iron supplements. All participants were provided of detailed explanation of the concept and design of study and gave a written informed consent before investigation. Before performing two different versions of WCE, all of the examinees first verified their normal position of duodenal papilla via esophagogastroduodenoscopy. Subjects were randomly assigned to each study group by using a computerized program. This study was examined and approved by the institutional review board of our institution.

Bowel preparation was done by drinking 4 liters of polyethylene glycol (PEG) solution early in the morning. Capsule was swallowed after verifying clear watery defecation, and examinees were allowed to take fluid 2 hours after the ingestion of capsule. Subjects were asked to stay in bed on a supine position during swallowing capsule and after start of capsule endoscopy for 3 hours. Examinees were allowed for ambulation thereafter. After removing the digital recording device, simple abdominal radiographs were taken from all participants to verify discharge of capsule from subjects' GI tract.

Diagnostic yield was evaluated in three aspects: (1) counting the number of digital photographic images with a view of duodenal papilla, (2) grading the maximum proportion of papillary outline in picture, and (3) scoring the resolution and brightness. In detail, papillary outline was graded as 3 (full), 2 (more than half), or 1 (less than half). If more than one image of papilla was captured from a subject, the maximum appearance of papilla was evaluated for scoring. Resolution and brightness were scored as 0 (low), 1 (medium), or 2 (high resolution or brightness), respectively, by six experienced gastroenterologists after watching a short video clip from two SB and two SB2 capsules, respectively. In order to blind the specific type of capsule from gastroenterologists, octagonal outline of SB2 capsule was hidden with black-colored paper onto the computer screen.

Quantitative data were reviewed by mean ± standard deviation (SD) or number and analyzed by the *t*-test. Categorical data were shown as a frequency (percentage) and handled by *χ* ^2^ test. Statistical analysis was done using a computerized statistical program (SPSS for Windows 12.0, SPSS Inc., Chicago, IL, USA). *P* values less than 0.05 were considered as a statistically significant result.

## 3. Results

The numbers of participants who prospectively enrolled and performed the SB and SB2 capsule endoscopy were 30 subjects, respectively, with indications (Table 1). Baseline characteristics such as sex or age were not statistically different. There was also no significant difference in terms of indication. All participants verified no capsule remaining in GI tract on simple abdominal radiographs. From each study group, two participants, respectively, showed suboptimal bowel preparation of each group, respectively, and these subjects were indicated to drink another 2 liters of PEG solution, resulting in satisfactory preparation of clear watery defecatory passage.

**Figure 1:**
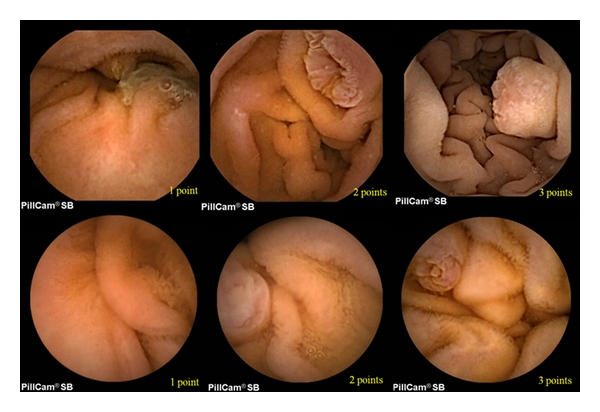
Representative images and scorings of papillary outline (marked on bottom right) of each capsule endoscopy (upper and lower panel for SB2 and SB, resp.). The octangular outline of SB2 images were hidden with black-colored paper in order not to be discerned by experienced gastroenterologists.

Table 2 summarizes the result of detection rate, number of images, and scoring of papilla of Vater of two study groups. SB and SB2 group revealed to have a papillary detection rate of 43.3% and 50.0%, respectively, with no significant statistical difference (*P* = 0.796). Number of photographic images containing the papilla also showed no statistical difference between two study groups (*P* = 0.571). The maximum point of evaluating the shape of papilla was collected, and scores marked from each participant were statistically similar between SB and SB2 study group. Some representative WCE images are illustrated on Figure 1.

Although SB2 capsule was superior to SB in terms of scoring of resolution (*P* = 0.001), scoring of brightness of SB and SB2 capsule revealed no significant difference (*P* = 0.165) (Table 3). In short, lack of improvement in brightness must be noted, whereas resolution exemplified significant difference between the two study groups.

## 4. Discussion

According to our investigation, PillCam SB2 capsule endoscopy failed to show major superiority over SB on observing duodenal papilla. The performance of SB2 was rather similar compared with the old version SB in several aspects as shown. Therefore, another technological step-up seems to be more important altogether with bowel preparation or visual obstacles (bubbles, bile, or debris), such as enhanced performance of photograph numbers per second. For example, one recent study addressed their result of digital capsule endoscopic images taken at a slower speed (i.e., 1 frame per second) does not deteriorate the overall performance of WCE, compared with the ordinary study speed of 2 frames per second [2]. A larger number of cases will verify our contention, but even as small participants, our study may give cautions to some physicians' blind optimism toward new technology and make them not to lose track of criticizing mere technological improvements. One recent investigation proved the limitation of faster image capture rates or two cameras on detecting a specific finding during WCE [3]. in contrast, some researchers refuted this study arguing that “double-headed” esophageal capsules and a faster viewing speed seems to retain a possible advantage [4]. Some investigators studied the performance of WCE [5] in response to our previous report [1], but omission of the exact appearance of papilla via EGD may have led to a lower detection rate of 10.4%. Although all subjects participated in various studies have a papilla, size and shape of papilla vary (see Figure 1) and sometimes a periampullary structural change such as diverticulum may influence the exact detection rate of papilla. Besides, there is very rapid transit of the capsule through the duodenum, and although it is usually clean without food or other debris, the papilla is missed.

It is also noteworthy that that WCE is not indicated for examining the duodenal papilla according to the current guidelines [6, 7]. The indications for the performance of capsule endoscopy (i.e., obscure gastrointestinal bleeding, established or suspected Crohn's disease, etc.) are already well known and being adopted in the clinical field; our purpose is not to add a new indication, but rather to use the papilla in order to compare the images taken by the two devices (Pillcam SB1 versus 2).

Since the advent on year 2000, WCE has changed the diagnosis and management of many diseases developing in small intestine. Many researchers studied the diagnostic power of this novel device, and investigators reported the incremental diagnostic yield in a spectrum of 39~90% in terms of obscure/occult gastrointestinal bleeding [8, 9]; one study group elucidated the estimated diagnostic yield of up to 91.1% [10]. However, the sensitivity and specificity presented on some previous studies lack confidence intervals [10]. That is, diagnostic accuracy should be presented with confidence interval in order not to make a considerable difference to a clinician's interpretation of the finding of a specific study [11]. For his reason, and also because of current shortage of established diagnostic accuracy, diagnostic yield is still used to represent the goodness of WCE instead of diagnostic accuracy.

While duodenal papilla may not be a good landmark because of its angular position and relatively swift transition of capsule in duodenum, it still can serve as the worst case of a possible miss rate because of similar shape and size with commonly missed lesions (angiodysplasias, small ulcers, etc.) [1]. Our results differ from previous reports studied with the old model [10] or the same new version alike we did [12]. We believe our study fulfills the role as a pilot study of the new version of device, prompting further studies investigating a larger number of subjects for clinical validation of this renewed apparatus.

## Figures and Tables

**Table 1:** Baseline characteristics and indications for wireless capsule endoscopy.

	SB (*n* = 30)	SB2 (*n* = 30)	*P* value
Sex (M/F)	14/16	15/15	NS
Age	46.1 ± 10.1	51.4 ± 9.2	NS
Healthy volunteers	3	2	NS
Obscure GIB	18	19	NS
Abdominal pain	5	6	NS
Chronic diarrhea	4	3	NS

(SB: small bowel capsule endoscope, SB2: small bowel capsule endoscope version 2, GIB: gastrointestinal bleeding, and NS: not significant)

*Values shown as mean ± SD or No.

**Table 2:** Results of wireless capsule endoscopy comparing SB and SB2 capsule.

	SB	SB2	*P* value
Detection rate (%)	43.3 (13/30)	50.0 (15/30)	0.796
No. of images of papilla	3.1 ± 1.1	3.1 ± 1.5	0.571
Image grading (max)			
3 points	4/13	4/15	
2 points	5/13	7/15	
1 point	4/13	4/15	

*Values shown as mean ± SD or No. max: maximum.

**Table 3:** Subjective scoring of resolution and brightness on SB and SB2 groups.

Parameters	SB (*n* = 12)	SB2 (*n* = 12)	*P* value
Resolution	0.93 ± 0.62	1.55 ± 0.40	0.001
Brightness	1.21 ± 0.70	1.57 ± 0.51	0.165

*Values shown as mean ± SD or No.
